# Validation of the 10‐Item Lower Extremity Functional Scale (LEFS‐10) for Individuals With Knee Osteoarthritis

**DOI:** 10.1002/pri.70153

**Published:** 2025-12-22

**Authors:** Daniella Dias de Oliveira, Aron Charles Barbosa da Silva, Jhonata Botelho Protazio, Gabriela Nascimento de Santana, Bruno Ruocco Verengue, Patrícia Gabrielle dos Santos, Almir Vieira Dibai‐Filho, Cid André Fidelis‐de‐Paula‐Gomes

**Affiliations:** ^1^ Postgraduate Program in Rehabilitation Sciences Nove de Julho University São Paulo Brazil; ^2^ Postgraduate Program in Physical Education Universidade Federal do Maranhão São Luís Maranhão Brazil

**Keywords:** chronic pain, knee osteoarthritis, measurement properties, musculoskeletal diseases, rehabilitation

## Abstract

**Background:**

Functional capacity assessment in knee osteoarthritis (KOA) is essential because it relates to pain, disability, and quality of life. Reliable, sensitive, and validated tools are needed to measure lower limb function.

**Purpose:**

To validate the Brazilian Portuguese version of the 10‐item Lower Extremity Functional Scale (LEFS‐10) for people with KOA.

**Methods:**

A cross‐sectional validation study involved 100 participants with KOA. The agreement between the 10‐item (LEFS‐10) and 20‐item (LEFS‐20) scales was examined using Spearman's correlation coefficient (rho) between the LEFS‐10 and the Western Ontario and McMaster Universities Osteoarthritis Index (WOMAC), the Numeric Pain Rating Scale (NPRS), and the World Health Organization Disability Assessment Schedule (WHODAS). Test‐retest reliability was measured with the intraclass correlation coefficient (ICC), the standard error of measurement (SEM), and the minimum detectable change (MDC). Internal consistency was assessed using Cronbach's alpha. Ceiling and floor effects were also evaluated.

**Results:**

Most individuals had right or bilateral KOA, with diagnosis duration and persistent pain exceeding 5 years. LEFS‐10 scores showed strong correlations with the original LEFS‐20 (rho = 0.92), NPRS (rho = −0.78), WOMAC pain (rho = −0.69), stiffness (rho = −0.67), physical function (rho = −0.70), and WHODAS (rho = −0.74). Internal consistency was adequate (Cronbach's alpha = 0.89). In a subsample of 50 participants, test–retest reliability was excellent (ICC = 0.98, 95% CI 0.97–0.99), with a SEM of 1.05 points (6.7%) and an MDC of 2.91 points (18.6%), with no ceiling or floor effects.

**Discussion:**

The LEFS‐10 demonstrates excellent concordance compared with LEFS‐20, good convergent validity, adequate internal consistency, high reliability, and favorable measurement error parameters, making it suitable for individuals with KOA.

## 1 Introduction

Assessing functional status is a crucial health indicator for individuals with knee osteoarthritis (KOA), especially as it declines due to loss of muscle strength, flexibility, and balance related to the condition. Factors such as the disease itself and inactivity can significantly affect their quality of life. Therefore, evaluating functional capacity in people with KOA is essential because it relates to pain levels, functional limitations, and overall quality of life (Darlow et al. 2025; Lozano‐Meca et al. 2024). As a result, employing more specific, accurate, sensitive, and validated assessment tools is vital for measuring lower limb function and overall capacity in individuals with KOA. Proper assessment forms the foundation for developing more effective management strategies, monitoring disease progression, and promoting self‐care (Lawford et al. 2024).

In this context, the Lower Extremity Functional Scale (LEFS) is a crucial assessment tool based on the World Health Organization's (WHO) model of disability and health. It provides valuable insights into functionality related to various activities involving lower limb function, as outlined in the International Classification of Functioning, Disability, and Health (ICF). As a result, the LEFS has been translated and culturally adapted into several languages, including Brazilian Portuguese, even for KOA populations (Metsavaht et al. 2012; Pereira et al. 2013). However, in recent years, studies conducted in multiple languages have led to the development of more refined and concise versions of the LEFS, demonstrating improved measurement properties and better reliability (Alnahdi 2018, 2021; Bravini et al. 2017; Fidelis‐de‐Paula‐Gomes et al. 2023; Repo et al. 2019). Among these adaptations, the 10‐item version of the LEFS for Brazilian Portuguese (LEFS‐10) is particularly noteworthy (Fidelis‐de‐Paula‐Gomes et al. 2023).

The development of the LEFS‐10 creates new opportunities for further research. While the initial study focused on validating its structure, the reliability of this shorter version should be tested with individuals who have specific lower limb functional conditions, such as knee osteoarthritis (KOA). Additionally, since the LEFS‐10 was created from responses to the LEFS‐20, this may have contributed to the strong correlation between the two versions. Therefore, additional analyses should evaluate concordance by applying the LEFS‐20 and LEFS‐10 separately (Fidelis‐de‐Paula‐Gomes et al. 2023).

From a clinical and research standpoint, validating the Brazilian Portuguese LEFS‐10 for individuals with KOA can offer several advantages over longer instruments. As a brief 10‐item scale with simple scoring, it reduces response burden, is easier to complete in busy clinical settings and for patients experiencing pain or fatigue, and enhances the feasibility of repeated assessments in both practice and research. In clinical studies, where participants often need to complete multiple questionnaires, shorter tools tend to improve adherence and response rates. By providing a concise yet accurate assessment of lower limb function in individuals with KOA, the LEFS‐10 can assist in creating personalized treatment plans and enable more efficient, flexible monitoring of disease progression. This may, in turn, improve self‐management and patients' awareness of their functional abilities, making the LEFS‐10 a practical and clinically valuable option for routine monitoring of lower limb function in this population. Therefore, this study aimed to validate the Brazilian Portuguese version of the LEFS‐10 for individuals with KOA.

## 2 Methods

### 2.1 Study Design and Ethical Considerations

Following the Declaration of Helsinki, this cross‐sectional methodological study validates the LEFS‐10 in individuals with KOA adhering to the Consensus‐Based Standards for the Selection of Health Measurement Instruments (COSMIN) (Prinsen et al. 2018). It was conducted in physical therapy clinics in São Paulo, Brazil, and received approval from the institution's research ethics committee. All participants provided written informed consent confirming their participation in the study.

### 2.2 Participants

The sample included 100 participants, meeting the minimum recommendation of seven participants per questionnaire item (Prinsen et al. 2018). Participants were required to have a clinical diagnosis of knee osteoarthritis, defined as symptomatic KOA lasting at least 3 months in individuals who reported knee pain for more than 3 months and scored higher than three on the Numeric Pain Rating Scale (NPRS). They also needed to have morning stiffness lasting less than 30 min, crepitus, bony tenderness, and no palpable warmth (Fitzgerald et al. 2015; Gholami et al. 2023; Alfredo et al. 2024). Additionally, participants had to be fluent, literate, and able to read and write in Brazilian Portuguese.

Individuals were excluded if their Mini‐Mental State Examination scores were below the cutoff values (23 or lower for those with higher education and 17 or lower for those with lower education) (Murden et al. 1991). Additionally, anyone who had undergone knee surgery or received intra‐articular steroid or hyaluronic acid injections during the study was also excluded. Furthermore, individuals who needed adrenocortical hormones or nonsteroidal anti‐inflammatory drugs for other medical conditions, such as cancer or diabetes, were excluded.

### 2.3 Data Collection

A physiotherapist recruited and assigned participants, while another administered the questionnaires. A third researcher conducted the data analysis. Participants completed the questionnaires independently during face‐to‐face interviews without a time limit. The physiotherapists, who had an average of 10 years of experience in physiotherapy and managing chronic musculoskeletal pain, underwent 3 months of training to ensure assessment accuracy.

Five questionnaires were administered. The NPRS, validated for Brazilian Portuguese, uses numbers from 0 to 10, where 0 signifies “no pain” and 10 indicates “the worst pain imaginable” (Ferreira‐Valente et al. 2011). The Western Ontario and McMaster Universities Osteoarthritis Index (WOMAC), also translated and validated for Brazilian Portuguese (Fernandes 2002), includes three domains: Pain, Stiffness, and Physical Function. Each item offers five response options (0–4), with higher scores indicating worse outcomes in each domain (Fernandes 2002). The World Health Organization Disability Assessment Schedule (WHODAS), validated for Brazilian Portuguese, assesses functionality through 12 items scored on a Likert scale from 1 to 5, where higher scores denote greater functional impairment (Grou et al. 2021).

Finally, the LEFS‐20 and LEFS‐10 were used to evaluate lower limb function. The LEFS‐20, translated into Brazilian Portuguese, includes 20 items, each scored on a 0 to 4 Likert scale, with higher scores indicating better function (Metsavaht et al. 2012; Pereira et al. 2013). The LEFS‐10, derived from the LEFS‐20, contains 10 items and offers a more detailed assessment of lower limb functional capacity (Fidelis‐de‐Paula‐Gomes et al. 2023).

Test‐retest reliability was evaluated in a subgroup of participants who completed the LEFS 10 twice, with a 7‐day interval between assessments.

### 2.4 Data Analysis

For sample characterization, quantitative data were summarized using the mean and standard deviation (SD), while qualitative data were reported as absolute numbers or percentages.

The Kolmogorov‐Smirnov test was used to assess data distribution, which was confirmed through histogram analysis. Since the data in the sample showed a non‐normal distribution, Spearman's correlation coefficient (rho) was employed. To evaluate concordance and convergent validity, Spearman's rho was used to measure the strength of the relationships among the WOMAC, NPRS, WHODAS, and LEFS‐10 domains.

For construct validity, the correlations were classified as follows: *r* < 0.30 indicates a weak correlation, *r* ≥ 0.30 and < 0.60 indicate a moderate correlation, and *r* ≥ 0.60 indicate a strong correlation. For concordance, the classifications were as follows: *r* ≥ 0.90 is considered excellent, *r* between 0.75 and 0.89 is regarded as good, *r* between 0.50 and < 0.74 is deemed moderate, and *r* < 0.50 is viewed as insufficient (Enderlein and Fleiss 1986; Terwee et al. 2012).

Internal consistency was assessed using Cronbach's alpha, with values from 0.70 to 0.95 considered adequate (Terwee et al. 2012).

Reliability was evaluated through a test‐retest method that included the intraclass correlation coefficient (ICC_2,1_), a 95% confidence interval (CI), standard error of measurement (SEM), and minimal detectable change (MDC). The following reference values were used to interpret ICC: low reliability (< 0.40), moderate reliability (0.40–0.75), substantial reliability (0.75–0.90), and excellent reliability (> 0.90). For SEM, the percentage of the total LEFS‐10 score was classified as very good (≤ 5%), good (> 5% and ≤ 10%), questionable (> 10% and ≤ 20%), and poor (> 20%). The formulas used were: SEM = SD × √(1− ICC) and MDC = 1.96 × vSEM × √2.38 (Barreto et al. 2022; de Vet et al. 2011).

This study examined the effects of the ceiling and floor. These effects were considered significant when 15% or more of the sample reached the minimum or maximum total score on the LEFS‐10 (Terwee et al. 2012).

The tested hypothesis was that the LEFS‐10 would demonstrate significant inverse correlations (≥ 0.50) with the WOMAC, WHODAS, and END domains, thereby supporting convergent validity, and that its agreement with the LEFS‐20 would be at least good.

## 3 Results

A total of 100 participants diagnosed with KOA were included (Table 1). Table 1 shows that most study participants were female, married, and had completed elementary school. They were characterized as overweight, physically inactive, undergoing physical therapy, and taking pain medication. Additionally, they had experienced pain for over 5 years and had more significant impairments in the right knee and bilaterally.

**TABLE 1:** Characteristics of the study participants (*n* = 100).

Variables	Percentage, median (SD)
Age (Years)	65.80 (7.26)
Weight (kg)	75.39 (10.84)
Height (*m*)	1,66 (0.99)
BMI (kg/m^2^)	27.53(4.46)
Sex	
Male	12%
Female	88%
Marital status	
Single	19%
Married	39%
Widower	23%
Divorced	19%
Education	
University complete	7%
High school complete	20%
Elementary school complete	73%
Physical activity	
Active	25%
Inactive	75%
Affected member	
Right	42%
Left	17%
Bilateral	41%
Diagnosis time (years)	8.14(5.16)
Duration of pain (years)	7.12(2.72)
Recurrent use of pain medication	
Yes	63%
No	37%
Physiotherapeutic treatment	
Yes	82%
No	18%
LEFS‐10 (0–40 points)	20.06 (8.47)
LEFS‐20 (0–80 points)	41.89 (17.51)
NPRS (0–10 points)	4.87 (1.66)
Womac	
Pain (0–20 points)	11.14 (3.39)
Stiffness (0–8 points)	4.04 (1.58)
Physical function (0–68 points)	34.95 (12.54)
Whodas (1–60 pontos)	25.78 (9.15)

Abbreviations: BMI, body mass index; kg, kilogram; LEFS‐10, lower extremity functional scale (high score = higher level of knee function); LEFS‐20, lower extremity functional scale (high score = higher level of knee function); m, meters; NPRS, numeric pain rating scale (high score = higher level of intensity of pain); SD, standard deviation; Whodas, World Health Organization Disability Assessment Schedule (high score = less level of knee function); Womac, western Ontario and McMaster University Osteoarthritis (high score = less level of knee function).

Table 2 shows the reliability, internal consistency, concordance, and convergent validity of the study participants. The concordance between LEFS‐10 and LEFS‐20 was rated as excellent. Convergence and concordance, based on the correlation between LEFS‐10 and the domains of WOMAC, NPRS, and WHODAS, were rated as good. Additionally, the negative correlations between WOMAC, NPRS, WHODAS, and LEFS‐10 confirmed the different structural compositions of the questionnaires. Furthermore, Table 2 includes the test‐retest reliability and internal consistency results, with values labeled as adequate for internal consistency according to Cronbach's alpha and excellent for ICC_2,1_. Moreover, the SEM percentage score was classified as good.

**TABLE 2:** Reliability, internal consistency, concordance, and convergent validity of the study participants.

Properties	Values	Classification
Concordance (*n* = 100)	0.92**	Excellent
Convergent validity (*n* = 100)		
NPRS (0–10 points, CI 95%)	−0.78** (−0.85, −0.69)	Good
Womac		
Pain (0–20 points, CI 95%)	−0.69** (−0.78, −0.57)	Good
Stiffness (0–8 points, CI 95%)	−0.67** (−0.76,−0.54)	Good
Physical function (0–68 points, CI 95%)	−0.70** (−0.78, −0.58)	Good
Whodas (1–60 pontos, CI 95%)	−0.74** (−0.81, −0.63)	Good
Internal consistency (*n* = 100)		
Cronbach's alpha	0.89	Adequate
Test‐retest reliability (*n* = 50)		
median (SD) −1	15.52 (7.39)	—
median (SD) −2	15.70(7.44)	—
ICC (CI 95%)	0.98 (0.97–0.99)	Excellent
SEM (points)	1.05	—
SEM (%)	6.72%	Good
MDC (points)	2.91	—
MDC (%)	18.62%	—

Abbreviations: CI 95%, confidence interval 95%; ICC, intraclass correlation coefficient; LEFS‐10, lower extremity functional scale (high score = higher level of knee function); LEFS‐20, lower extremity functional scale (high score = higher level of knee function); MDC, minimal detectable change; NPRS: numeric pain rating scale (high score = higher level of intensity of pain); SD, standard deviation; SEM, standard error of measurement; Whodas, World Health Organization Disability Assessment Schedule (high score = less level of knee function); Womac, western Ontario and McMaster University Osteoarthritis (high score = less level of knee function).

^**^ Significant correlation (*p* < 0.01).

Only three individuals reached the minimum score, while one person attained the maximum score on the LEFS‐10. As a result, no ceiling or floor effects were observed.

## 4 Discussion

Our results show that the LEFS‐10 has excellent concordance and strong convergent validity with the WOMAC, NPRS, and WHODAS domains. It also exhibits adequate internal consistency, with reliability measures rated as excellent (ICC_2,1_) and good (%SEM). Based on these findings, the study hypothesis was confirmed, establishing that the LEFS‐10 is a valid and reliable tool for use in individuals with KOA.

The LEFS‐10 showed correlation values well above 0.70, as recommended by COSMIN (Terwee et al. 2012). This indicates that its 10‐item structure closely aligns with the original 20‐item version while providing the benefit of a faster, more targeted assessment. Regarding construct validity, the correlation values between the LEFS‐10 and tools measuring pain intensity, pain behavior, stiffness, physical function, and functionality exceeded the suggested thresholds, ranging from 0.30 to 0.50 (Terwee et al. 2012; Prinsen et al. 2018). These values were even higher than those reported in the 15‐item Arabic version, highlighting the LEFS‐10's ability to capture a broad range of signs and symptoms despite having fewer items (Alnahdi 2021).

Regarding internal consistency, the LEFS‐10 showed lower values compared to the 15‐item Arabic version (Cronbach's alpha = 0.93) (Alnahdi 2021) and the Dutch version used with individuals experiencing KOA (Cronbach's alpha = 0.96) (Hoogeboom et al. 2012). However, despite this lower value, the LEFS‐10 demonstrated a suitable structure, remaining within the recommended reference range (0.70–0.95) (Hoogeboom et al. 2012; Pan et al. 2014). This indicates that its items are correlated, homogeneous, and most importantly, not redundant, as the values did not exceed 0.95, the cutoff for potential item redundancy in the instrument (Alnahdi 2018, 2021; Hoogeboom et al. 2012).

A measure inherently connected to ICC is the SEM, which is derived from the standard deviation and test‐retest reliability (ICC values) (Fernandes 2002). Higher ICC values indicate less variability in test‐retest results and therefore more consistent SEM values. This was evident with the LEFS‐10, which, despite having higher SEM values than other versions involving individuals with KOA, was still classified as good (Alnahdi 2018, 2021; Williams et al. 2012). It is important to recognize that both SEM and MDC values may have been affected by the interventions or therapeutic resources used by the participants during the test‐retest period. Over 80% of the sample was receiving physical therapy, and more than 60% were taking pain medications, which could have influenced the results.

Therapeutic interventions may have affected both SEM and MDC values, as over 80% of participants received physical therapy and more than 60% used pain medication during the test‐retest period. When using the LEFS‐10, clinicians, researchers, and individuals with KOA should account for an error margin of 1.05 points for a single assessment. A meaningful change is indicated by score differences of 3 points or more, which helps track functional changes over time. Therefore, the LEFS‐10 is a self‐reported functional assessment tool suitable for clinical and research settings. In addition to its shorter completion time, it improves the relevance of items to the individual's clinical condition. With fewer items, the LEFS‐10 may offer a more accurate self‐assessment of functionality, supporting the development of self‐management strategies for daily activities rather than relying solely on standardized clinical tests such as sit‐to‐stand or walking tests.

This study has several limitations that should be acknowledged. First, the convenience sampling method might not accurately represent the broader KOA population. The sample included only individuals receiving physical therapy, which could have influenced their self‐reported responses due to the attention and treatments provided. Second, this was a cross‐sectional validation study, so we did not evaluate responsiveness, and longitudinal studies are needed to establish the sensitivity of LEFS 10 scores to clinical change. Additionally, some analyses were not performed, leaving opportunities for future research. Specifically, reproducibility assessments comparing differences between evaluators were not conducted. While traditional methods were used to evaluate the measurement properties based on self‐reports, future studies could utilize modern techniques such as Rasch analysis. Lastly, the LEFS 10 showed excellent reliability and acceptable measurement error. However, the MDC percentage of 18.62% indicates that relatively large changes are needed to confidently confirm a true change at the individual level. Smaller score changes should be interpreted cautiously in clinical practice and ideally considered together with other clinical information and repeated measurements.

The strong correlation between the LEFS 10 and the original LEFS 20 indicates that the shorter version retains essential information about lower limb function relevant to individuals with KOA. Therefore, the LEFS 10 seems to preserve the core construct measured by the full version, offering practical benefits such as shorter completion times and reduced response burden in clinical and research environments with many patients, where completing multiple questionnaires is common. This supports using the LEFS 10 as a more practical and efficient option when quick assessment is needed, without significantly compromising the overall score's information.

In conclusion, our findings indicated that the LEFS‐10 showed no ceiling or floor effects, demonstrated adequate internal consistency, excellent test‐retest reliability, and a good measurement error. Additionally, its correlation patterns with other outcome measures matched the predefined hypotheses, thus supporting its construct validity as a functional assessment tool for individuals with KOA.

## 5 Implications of Physiotherapy Practice

The LEFS‐10 is a self‐reported outcome measure suitable for both clinical and research settings to evaluate functional status in individuals with KOA. Its brief format shortens completion time and improves item relevance to the clinical condition. By emphasizing patient‐reported functional abilities in daily life rather than standardized test tasks (e.g., sit‐to‐stand or walking tests), the LEFS‐10 may better support personalized self‐management strategies.

## Funding

The authors have nothing to report.

## Ethics Statement

Approval for the study was obtained from the Local Ethics Committee of a private University in São Paulo, Brazil. Ethical approval number—CAAE: 70415023.1.0000.5511.

## Consent

Informed consent was obtained from all subjects involved in the study.

## Conflicts of Interest

The authors declare no conflicts of interest.

## Data Availability

The data that support the findings of this study are available from the corresponding author upon reasonable request.
